# Protective effect of *Chlorella vulgaris* on sub-chronic hepatic and testicular toxicity induced by acetamiprid in rats

**DOI:** 10.3389/fvets.2026.1782904

**Published:** 2026-04-24

**Authors:** Huda M. Fouda, Tarek A. Yosef, Samia M. Abd El-Rheem, Essam A. Almadaly, Hanaa M. Hegazy

**Affiliations:** 1 Department of Forensic Medicine, Faculty of Veterinary Medicine, Kafrelsheikh University, Kafrelsheikh, Egypt; 2 Department of Theriogenology, Faculty of Veterinary Medicine, Alexandria University, Alexandria, Egypt; 3 Department of Theriogenology, Faculty of Veterinary Medicine, Kafrelsheikh University, Kafrelsheikh, Egypt

**Keywords:** acetamiprid, *albino* rats, *Chlorella vulgaris*, comet assay, liver, testis

## Abstract

Acetamiprid (ACMP) is a widely used neonicotinoid insecticide that has been linked to oxidative stress-induced toxicity and physiological changes in mammals. This study investigates ACMP-induced hepatic and testicular toxicity in rats and the potential protective role of *Chlorella vulgaris* (*C. vulgaris*), a green microalga used as an antioxidant against ACMP-induced toxicity. Thirty-two male rats were allocated into four groups of eight each, as follows: (1) The CTRL group received only water; (2) The ACMP group received 21.7 mg/kg body weight (BW) of ACMP; (3) The *C. vulgaris* group received 150 mg/kg BW; and (4) The combined group received the same previously mentioned doses of ACMP and *C. vulgaris*. All treatments were given daily by oral gavage for 9 weeks. After euthanasia, testicular sperm were recovered to evaluate sperm characteristics. Hepatic and testicular samples were collected for biochemical analysis, comet assay, and histopathological examination. The ACMP group had lower sperm characteristics and enzymatic antioxidants in hepatic and testicular tissues, concurrent with a significant elevation of hepatic and testicular malondialdehyde. Moreover, the ACMP group revealed a significant increase in DNA damage with marked histopathological changes in the liver and testis. On the contrary, co-administration of *C. vulgaris* with ACMP alleviated the adverse effects of ACMP in the liver and testis and restored most of the measured parameters to levels comparable to those of the CTRL group. In conclusion, this apparent protective role of *C. vulgaris* against ACMP-induced toxicity in rats may be due to its potent antioxidant properties.

## 1 Introduction

Pesticides are one of the most common pollutant groups worldwide. They are the chemical formulation increasingly used in agriculture, animal husbandry, and public health operations to control insect-transmitted diseases and cause environmental pollution (1). Neonicotinoid insecticides are among the most widely used insecticides applied to crops, having been the world's largest-selling insecticides for many years (2). It is synthetic analogs of nicotine that act as agonists to the acetylcholine molecule, blocking its binding to postsynaptic cell receptors, which excite neurons and result in paralysis and death. The postsynaptic nicotinic acetylcholine receptors (nAChRs) are the unique site of their binding (3). Since the mammalian nervous system (a4, b2), human testis (a5, b4), prostate (a5), mouse testis and sperm (a7) all express distinct subtypes of nAChRs, these organs are thought to be the primary targets of neonicotinoids (4–6).

One of the neonicotinoid insecticides is acetamiprid (ACMP). It is a second-generation neonicotinoid insecticide that was initially commercialized in Japan in 1995 by Nippon Soda (Nippon Soda Co Ltd., Tokyo, Japan), mainly for foliar applications, and direct soil uses are restricted (7). ACMP is an organic compound known as the International Union of Pure and Applied Chemistry as N-[(6-chloro-3-pyridyl) methyl]-N′-cyano-N-methyl-acetamidine and comprises a heterocyclic ring (the 6-chloro-3-pyridylmethyl moiety), which is one of the most effective insecticides for the protection of crops in the world (8). ACMP is systemic in action and controls sucking insects (9). It has high water solubility, high soil permeability, and the potential to be toxic to humans. Agricultural goods, fruits, food, water, and soil have all been shown to have residues of ACMP and its metabolites, which make the non-targeted organisms more susceptible to their exposure (10). A growing body of evidence has established that exposure to ACMP results in its accumulation and increases the levels of its metabolites in several organs, causing organ dysfunction (11). Various studies have shown that ACMP exposure mediates hepatotoxicity (12), neurotoxicity (13), reproductive toxicity (14), cytotoxicity, and genotoxicity (8, 15).

In natural conditions, a balance exists between the production and removal of free radicals, which can cause serious damage to cells (16). Most cells can deal with a mild percentage of oxidative stress. Excessive free radicals lead to lipid peroxidation (LPO), DNA damage, and depletion of antioxidant reserves (17). Earlier studies have revealed that ACMP causes an imbalance between oxidants and the antioxidant defense system inside the body, leading to oxidative stress (13, 18) by producing reactive oxygen species (ROS) (19). ROS include non-free radical species, such as hydrogen peroxide (H_2_O_2_) and singlet oxygen (^1^O_2_), as well as free radicals like superoxide anion radicals (${\text{O}}_{2}^{-}$) and hydroxyl radicals (OH•), which can easily initiate the peroxidation of membrane lipids, leading to the accumulation of lipid peroxides.

The reproductive system is one of the most vulnerable parts of an organism to environmental and occupational pollutants, especially pesticides. Insecticides have been shown to induce oxidative stress in the testis, resulting in damaged spermatozoa, altered spermatogenesis, and impaired Sertoli or Leydig cell function, as well as disrupting endocrine function at any stage of hormone regulation (20). Najafi et al. (21) observed obvious alterations in semen quality, testosterone levels, and testicular architecture in mature male rats treated with imidacloprid, which is one of the neonicotinoids. Similarly, Zhang et al. (19) reported that the dose of 30 mg ACMP/kg body weight (BW) induced reproductive toxicity, biochemical changes, and oxidative stress in mice.

The liver has been considered the most important organ targeted by the toxic effects of xenobiotics such as pesticides. Pesticide exposure has been linked to oxidative stress (22). The acute toxicity data indicate that ACMP is toxic via the oral route in mammals, with DL_50_: 217 mg/kg for male rats. It is rapidly and almost completely absorbed, widely distributed into the tissues, and found at the highest concentrations in the gastrointestinal tract, adrenal gland, liver, and kidney (9, 23). Sub-chronic ACMP exposure alters serum biochemical markers, particularly hepatic enzymes (23). In addition, sub-chronic ACMP exposure reduces the activity of antioxidant enzymes in the liver, including superoxide dismutase (SOD), catalase (CAT), and glutathione peroxidase (GPx). ACMP-induced oxidative stress causes significant oxidative damage in hepatic cells, leading to cell malfunction with subsequent cell death (19, 24).

ACMP may cause DNA damage by producing ROS (25). According to Yao et al. (26), ACMP temporarily raised the levels of CAT and SOD in three different bacterial species. When the activity of these enzymes declines, more superoxide and hydrogen peroxide are produced, leading to the formation of hydroxyl free radicals that can disrupt DNA strands (27). The exact genotoxicity mechanism of ACMP is currently unknown. Genotoxic substances can interact with DNA in either an intercalative or non-intercalative manner. Intercalation usually induces conformational changes in DNA. Jie et al. (28) revealed that ACMP may interact with single-stranded or double-stranded DNA in a non-intercalative manner. It has become necessary to evaluate the potential genotoxic effect of ACMP on living organisms due to its increasing widespread use in the world.

Algae, a novel food with nutritional benefits, is currently used in medicine for a variety of uses. Furthermore, algae provide a rich supply of natural bioactive compounds with several biological activities (29). One of the algae that has the active component is the microalgae *Chlorella vulgaris* (*C. vulgaris*), which is the most important member of the *Chlorellaceae* family from the *Chlorophyta* group.

*C. vulgaris* is a freshwater, single-cell alga and has the highest amount of chlorophyll of any plant known. It is also a nutrient-dense superfood that contains 18 amino acids, including the essential ones, and 60% protein (30). It is also rich in many other valuable substances, including proteins, carbohydrates, vitamins, and antioxidants (31). It contains several antioxidants, such as β-carotenoids, chlorophyll, ascorbic acid, α-tocopherol, phenolic compounds, astaxanthin, flavonoids, and trace minerals including zinc, copper, and magnesium, which are necessary for antioxidant metalloenzyme activity (31, 32). The higher content of carotenoids in microalgae has shown not only antioxidant properties but also anti-inflammatory properties (33). Moreover, *C. vulgaris* is rich in linoleic and α-linolenic acids, which act as precursors of n-3 polyunsaturated fatty acids (PUFAs) such as eicosapentaenoic acid and docosahexaenoic acid (34). According to Cha et al. (35), *C. vulgaris* extract included 15 different antioxidants.

Almutairi et al. (36) found that *C. vulgaris* protected the liver and kidney against cisplatin-induced damage in rats. Furthermore, *C. vulgaris* supplementation improved rabbit performance by reducing oxidative stress and enhancing antioxidant enzyme activity (37). Also, it protects mice and rats' livers and reproductive systems against carbon tetrachloride and doxorubicin-induced damage (38, 39). Similarly, *C. vulgaris* protected brain tissue in rats (40). As a result, this study investigates the toxicological effects of ACMP on sperm characteristics, hepatic and testicular tissues, and the possible protective role of *C. vulgaris* against ACMP-induced toxicity in male rats was also investigated.

## 2 Materials and methods

### 2.1 Materials

The commercial product that was used as a source of ACMP was CRSTPRID 40%, a water-soluble powder obtained from Crest Company for chemicals in Egypt. Its chemical formulation is N-[(6-chloro-3-pyridyl) methyl]-N′-cyano-N-methyl-acetamidine. Dried *C. vulgaris* powder was obtained from the Feed Crop Department, Agricultural Research Center, Giza, Egypt. Kits of malondialdehyde (MDA), CAT, SOD, and reduced glutathione (GSH) in the liver and testis tissues homogenate were obtained from Bio-diagnostic Company, Giza, Egypt. Phosphate-buffered saline (PBS) was obtained from Thermo Fisher Scientific Company, USA. All materials were of analytical grade and obtained from Merck, Sigma (Rahway, New Jersey), and Thermo Fisher Scientific (Third Avenue, Waltham, MA) Companies, USA.

### 2.2 Animals and experimental design

A total of 32 male *albino* rats of the *Wistar* strain, weighing 130 ± 30 g, were used in this study. The rats were obtained from the laboratory animal house of the Modern Veterinary Office, Giza, Egypt. Rats were reared within polyethylene cages in a well-ventilated house at 22 ± 3 °C, 50%−60% relative humidity, and a 12/12 h light and dark cycle. Rats had free access to fresh water and were fed a well-balanced ration, and were kept for 2 weeks before the onset of the experiment until the animals attained a body weight of ≈190 ± 30 g.

The animals were randomly divided into four groups, 8 rats/each as follows: (1) This group was designed as the control (CTRL) group rats was administered with distilled water only; (2) The ACMP group in which each rat received 21.7 mg/kg BW of ACMP according to Chakroun et al. (41); (3) The *C. vulgaris* group in which rats received 150 mg/kg BW of *C. vulgaris* (42); (4) The combined group (ACMP + *C. vulgaris*) was treated with 21.7 mg/kg BW of ACMP + 150 mg/kg BW of *C. vulgaris*. The route of administration of all treatments was ingestion by oral gavage for nine successive weeks.

### 2.3 Tissue collection

At the end of the experiment (24 h after the last administration), rats were fasted overnight, anesthetized using diethyl ether, and euthanized by cervical dislocation. A sterile scalpel blade was used to make a mid-caudal (abdominal) incision for post-mortem examination immediately after euthanasia. The liver and one testis of each rat were collected, washed with normal saline, and blotted over a piece of filter paper before being sliced into three portions for analysis. The first portion for the oxidant and antioxidants analysis was kept in PBS at −20 °C until analysis. The second portion was stored in PBS (pH 7.4) at −80 °C until examination for the comet assay. The third portion was kept in 10% formalin for histopathological examination (HPE).

### 2.4 Sperm characteristics

Sperm cells were recovered from the other testis and epididymis by the slicing method (43) using warm (37 °C) sodium citrate dihydrate 2.9%. The sperm suspension was examined for the following:

#### 2.4.1 Sperm motility

Sperm motility was evaluated using the method described by Aly and Azhar (44). A 10 μl aliquot of sperm suspension was spotted on a prewarmed (37 °C) glass slide, covered with a warm cover slip (18 mm × 18 mm), and examined under a phase-contrast ( × 400) microscope. The proportions of progressive (PM: movement in straight lines or large circles) and total motility (TM: all forms of sperm movements, including straight, circular, and oscillatory) were determined.

#### 2.4.2 Plasma membrane integrity

The hypoosmotic swelling test (HOST) was used to determine the sperm plasma membrane integrity according to the methodology of Almadaly et al. (45). Briefly, 100 μl of sperm suspension was diluted with 1,000 μl of hypoosmotic solution (7.35 g Sodium citrate dihydrate and 1.351 g Fructose dissolved in 100 ml Milli-Q water, resulting in an osmolarity of 150 mosm/L), and incubated at 37 °C for 30 min. After incubation, 2 μl of sperm suspension was spotted onto a glass slide, covered with a cover slip, and examined under phase-contrast microscopy with a magnification lens of × 400. Upon examination, the sperm showing swollen or curled tails (HOST-positive) were considered membrane-intact, while those lacking this feature were considered membrane-damaged. To ensure accurate results, any sperm cells exhibiting abnormal tail morphology were counted before HOST and subtracted from the % of HOST-positive spermatozoa to obtain the true % of HOST-positive spermatozoa (45).

#### 2.4.3 Sperm viability

Sperm cell viability was evaluated in an eosin–nigrosin–stained semen smear. An aliquot (2 μl) of sperm suspension was mixed with 20 μl prewarmed eosin-nigrosin stain, smeared on a glass slide, dried on a warming plate, and examined under × 400 magnification of a bright-field microscope. Upon examination, sperm cells with unstained sperm heads were considered viable, while sperm cells with pink-stained heads were counted as nonviable cells (46).

#### 2.4.4 Sperm cell concentration (SCC)

An aliquot of sperm suspension (10 μl) was mixed with 1,000 μl of sperm counting medium [SCM; 0.9% NaCl, 0.1% formaldehyde, 10 mM ethylene diamine tetraacetic acid (EDTA), and 0.1% polyethylene glycol]. From this mixture, transfer 12.5 μl into the hemocytometer counting chamber. Count sperm cells under a phase contrast microscope at × 400 magnification (47).

### 2.5 Oxidant and antioxidant analysis

The determined portion from the liver and testis for oxidant and antioxidant analysis was thawed and perfused with PBS (pH 7.4) containing 0.16 mg/ml heparin before dissection to remove any red blood cells and clots. The dissected tissue was homogenized in 5–10 ml of 50 mM potassium phosphate of pH 7.5 per gram of tissue. The homogenate was centrifuged at 4,000 rpm for 15 min at 4 °C, and the supernatant was recovered and stored at – 80 °C until analysis.

The MDA (LPO biomarker) was determined colorimetrically at the wavelength of 534 nm according to Satoh (48). The activity of CAT was determined colorimetrically at the wavelength 510 nm according to Aebi (49), and the activity of SOD was determined colorimetrically at the wavelength 560 nm according to Nishikimi et al. (50). Also, the activity of GSH was determined colorimetrically at the wavelength of 405 nm according to Beutler et al. (51).

### 2.6 Comet assay

The dissected portions of both liver and testis were thawed and placed in a tube that contained 1 ml of cold PBS with 20 mM EDTA and 10% Dimethyl sulfoxide (DMSO). These portions were minced into fine pieces, allowed to settle down for a while, and 10 μl of cell sediment was mixed with 90 μl of low-melting-point agarose at 37 °C. The purpose of DMSO is to prevent LPO during tissue processing, while the ideal cell count is around ≈10,000 cells per slide. Then spread on a base slide that has been pre-coated with a layer of 1% normal melting point agarose at 4 °C for 10–15 min, then the slides were placed in Lysis solution (2.5 mol/L NaCl, 100 mmol/L Na_2_EDTA, 10 mmol/L Tris base, and around ≈8 g NaOH) and allow the mixture to dissolve for about 20 min before adjusting the pH to 10. Finally, add a freshly prepared 1% Triton X-100 and 10 % DMSO and incubate for 2 h at 4 °C. Arrange slides side by side on the horizontal gel box near one end. Fill the buffer reservoirs with freshly prepared electrophoresis buffer (300 mmol/L NaOH/1 mmol/L Na_2_EDTA) of pH >13 until the liquid level fully covers the slides. Allow slides to remain in the alkaline buffer for 20 min at 4 °C to allow DNA to unwind and alkali-labile damage to be expressed.

The power supply was turned on at 24 volts, and the current was set to 300 milliamperes. The slides were run for 30 min. The slides were washed with neutralization buffer (0.4 mol/L Tris, pH 7.5) three times. Then stained with 20 μg/ml Ethidium Bromide (EtBr) for 5 min and then dipped in chilled distilled water to remove excess stain. Afterwards, the EtBr-stained slides were covered with a coverslip, and the DNA was examined under a fluorescence microscope (Olympus BX51, Tokyo, Japan) with a × 400 magnification. For each cell, the length of DNA (tail length), the percentage of DNA in the tail, and the tail moment were estimated. DNA damage was measured using image analysis comet score (AutoComet.com, Ver1.5) (52).

### 2.7 Histopathological examination

The hepatic and testicular tissues were fixed with 10% neutral buffered formalin and then prepared using the standard paraffin embedding techniques. Briefly, the fixed tissues were dehydrated using a sequence of increasing ethyl alcohol concentrations. Dehydrated tissues were cleaned with xylene, fixed in paraffin wax, cut into 5 μm slices, and stained with hematoxylin and eosin (H&E) (53). The stained slices were examined under a light microscope (Leica DFC295, Wetzlar, Germany) for the histological changes in the liver and testis in the four experimental groups.

### 2.8 Statistical analysis

The results were expressed as mean ± standard error of the mean (SEM). The statistical analysis was performed using SPSS 20.0 (IBM SPSS Statistics, Egypt). One-way ANOVA followed by Duncan's multiple range test (54) was used to detect the differences among treatments at a significance level of *P* < 0.05. *The Shapiro-Wilk* test and the *Levene* test were used, respectively, to check the normality and homogeneity of variance of the data. Meet the normal distribution.

## 3 Results

### 3.1 Sperm characteristics

#### 3.1.1 Sperm motility

The ACMP group showed a significant (*P* < 0.05) decline in the proportions of TM (19.93 ± 0.41% vs. 46.71 ± 0.35%) and PM (10.10 ± 0.25 vs. 18.43 ± 0.21%) when compared to the CTRL group. Co-administration of ACMP with *C. vulgaris* showed a significant increase in the proportions of TM (36.60 ± 0.37%) and PM (14.24 ± 0.25%) when compared to the ACMP group. *C. vulgaris*-treated rats had the greatest % of TM (57.05 ± 0.35%) and PM (20.72 ± 0.23%) when compared to the CTRL group, as shown in Table 1.

**Table 1:** Effect of oral administration of ACMP (21.7 mg/kg BW) and/or *C. vulgaris* (150 mg/kg BW) on the sperm characteristics of male rats (*n*/group = 8).

Experimental groups	TM (%)	PM (%)	Plasma membrane integrity (%)	Viability (%)	SCC ( × 10^6^/ml)
CTRL	46.71 ± 0.35^b^	18.43 ± 0.21^b^	41.26 ± 0.22^b^	46.92 ± 0.38^b^	30.45 ± 0.38^b^
ACMP	19.93 ± 0.41^d^	10.10 ± 0.25^d^	12.50 ± 0.42^d^	21.17 ± 0.27^d^	10.41 ± 0.34^d^
*C. vulgaris*	57.05 ± 0.35^a^	20.72 ± 0.23^a^	47.78 ± 0.40^a^	62.54 ± 0.18^a^	40.38 ± 0.33^a^
ACMP + *C. vulgaris*	36.60 ± 0.37^c^	14.24 ± 0.25^c^	36.13 ± 0.26^c^	39.84 ± 0.36^c^	27.66 ± 0.14^c^

Within the same column, means bearing different superscripts were significant at *P* < 0.05. Results are expressed as mean ± SEM. TM, total motility; PM, progressive motility; SCC, sperm cell concentration; CTRL, control; ACMP, acetamiprid; *C. vulgaris, Chlorella vulgaris*; ACMP + *C. vulgaris*, acetamiprid + *Chlorella vulgaris*.

#### 3.1.2 Plasma membrane integrity

The plasma membrane integrity was significantly different among the four groups, where the ACMP group had the lowest proportion of intact plasma membrane (12.50 ± 0.42%) in comparison to the CTRL (41.26 ± 0.22%), *C. vulgaris* (47.78 ± 0.40%), and ACMP + *C. vulgaris* (36.13 ± 0.26%) groups, as shown in Table 1.

#### 3.1.3 Sperm viability

Table 1 revealed that the sperm cell viability was significantly (*P* < 0.05) lower in the ACMP (21.17 ± 0.27%) group than in the CTRL (46.92 ± 0.38%), *C. vulgaris* (62.54 ± 0.18%), and *C. vulgaris* + ACMP (39.84 ± 0.36%) groups.

#### 3.1.4 Sperm cell concentration

The obtained results showed a significant (*P* < 0.05) differences in the SCC among groups, where it was lower in the ACMP (10.41 ± 0.34 × 10^6^/ml*)* group than the CTRL (30.45 ± 0.38 × 10^6^/ml), *C. vulgaris* (40.38 ± 0.33 × 10^6^/ml), and ACMP + *C. vulgaris* (27.66 ± 0.14 × 10^6^/ml) groups with significant difference among all groups as presented in Table 1.

### 3.2 Oxidant and antioxidant in the liver and testis

#### 3.2.1 MDA level

The sub-chronic administration of ACMP showed a significant (*P* < 0.05) increase in MDA level in the liver (49.79 ± 0.25 vs. 30.05 ± 0.26 nmol/mg) and testis (10.83 ± 0.10 vs. 4.71 ± 0.06 nmol/mg) when compared to the CTRL group. On the other hand, there was a significant reduction in MDA level in the ACMP co-administered with *C. vulgaris* group in the liver (34.24 ± 0.20 nmol/mg) and testis (5.51 ± 0.10 nmol/mg) when compared to the ACMP group. In *the C. vulgaris* group, there was a significant (*P* < 0.05) reduction in MDA level in liver (22.88 ± 0.28 nmol/mg) and testis (3.40 ± 0.16 nmol/mg) when compared to the CTRL group (Tables 2, 3).

**Table 2:** Effect of oral administration of ACMP (21.7 mg /kg BW) and/or *C. vulgaris* (150 mg /kg BW) on the oxidant and antioxidant activities in the liver of male rats (*n*/group = 8).

Experimental groups	MDA (nmol/mg)	CAT (U/g)	SOD (U/g)	GSH (mg/g)
CTRL	30.05 ± 0.26^c^	59.31 ± 0.18^b^	61.34 ± 0.17^b^	14.81 ± 0.13^b^
ACMP	49.79 ± 0.25^a^	29.14 ± 0.23^d^	41.27 ± 0.23^d^	5.64 ± 0.13^d^
*C. vulgaris*	22.88 ± 0.28^d^	67.26 ± 0.20^a^	62.53 ± 0.19^a^	16.72 ± 0.13^a^
ACMP + *C. vulgaris*	34.24 ± 0.20^b^	52.59 ± 0.34^c^	57.10 ± 0.26^c^	12.59 ± 0.14^c^

Within the same column, means bearing different superscripts were significant at *P* < 0.05. Results are expressed as mean ± SEM. CTRL, control; ACMP, acetamiprid; *C. vulgaris, Chlorella vulgaris*; ACMP + *C. vulgaris*, acetamiprid + *Chlorella vulgaris*; MDA, malondialdehyde; CAT, catalase; SOD, superoxide dismutase; GSH, reduced glutathione.

**Table 3:** Effect of oral administration of ACMP (21.7 mg /kg BW) and/or *C. vulgaris* (150 mg /kg BW) on oxidant and antioxidant parameters in the testis of male rat (*n*/group = 8).

Experimental groups	MDA (nmol/mg)	CAT (U/g)	SOD (U/g)	GSH (mg/g)
CTRL	4.71 ± 0.06^c^	12.71 ± 0.06^b^	12.50 ± 0.14^b^	10.42 ± 0.13^b^
ACMP	10.83 ± 0.10^a^	5.70 ± 0.13^d^	8.45 ± 0.12^d^	5.64 ± 0.10^d^
*C. vulgaris*	3.40 ± 0.16^d^	14.61 ± 0.13^a^	14.61 ± 0.13^a^	11.79 ± 0.07^a^
ACMP+ *C. vulgaris*	5.51 ± 0.10^b^	11.21 ± 0.23^c^	11.32 ± 0.11^c^	9.63 ± 0.16^c^

Within the same column, means bearing different superscripts were significant at *P* < 0.05. Results are expressed as mean ± SEM. CTRL, control; ACMP, acetamiprid; *C. vulgaris, Chlorella vulgaris*; ACMP + *C. vulgaris*, acetamiprid + *Chlorella vulgaris*; MDA, malondialdehyde; CAT, catalase; SOD, superoxide dismutase; GSH, reduced glutathione.

#### 3.2.2 CAT activity

The ACMP group had the lowest CAT activity in hepatic (29.14 ± 0.23 U/g) and testicular (5.70 ± 0.13 U/g) tissues compared to their counterparts in the CTRL group (59.31 ± 0.18 U/g and 12.71 ± 0.06 U/g). Co-administration of ACMP + *C. vulgaris* showed a significant increase in CAT activity in hepatic (52.59 ± 0.34 U/g) and testicular (11.21 ± 0.23 U/g) tissues compared with the ACMP group. CAT activity was the greatest in *C. vulgaris* in either hepatic (67.26 ± 0.20 U/g) or testicular (14.61 ± 0.13 U/g) tissue in comparison with other experimental groups, as illustrated in Tables 2, 3.

#### 3.2.3 SOD activity

The results obtained revealed that there was a significant reduction in SOD activity in the ACMP group in the liver (41.27 ± 0.23 vs. 61.34 ± 0.17 U/g) and testis (8.45 ± 0.12 vs. 12.50 ± 0.14 U/g) compared to the CTRL group. Co-treatment of ACMP + *C. vulgaris* resulted in a significant increase in SOD activity in the liver (57.10 ± 0.26 U/g) and testis (11.32 ± 0.11 U/g) when compared to the ACMP group. *C. vulgaris* group had the highest SOD activity among all groups, either in liver (62.53 ± 0.19 U/g) or testis (14.61 ± 0.13 U/g), as presented in Tables 2, 3.

#### 3.2.4 GSH concentration

Oral administration of ACMP showed a significant (*P* < 0.05) decrease in GSH concentration in hepatic (5.64 ± 0.13 vs. 14.81 ± 0.13 mg/g) and testicular (5.64 ± 0.10 vs. 10.42 ± 0.13 mg/g) tissues in comparison with the CTRL group. However, co-administration of ACMP with *C. vulgaris* resulted in significant improvement of GSH concentration in hepatic (12.59 ± 0.14 mg/g) and testicular (9.63 ± 0.16 mg/g) tissues when compared to the ACMP group. In *C. vulgaris*, there was an elevation of GSH concentration in hepatic (16.72 ± 0.13 mg/g) and testicular (11.79 ± 0.07 mg/g) tissues when compared to the CTRL group (Tables 2, 3).

### 3.3 Comet assay

#### 3.3.1 Liver

**Figure 1:**
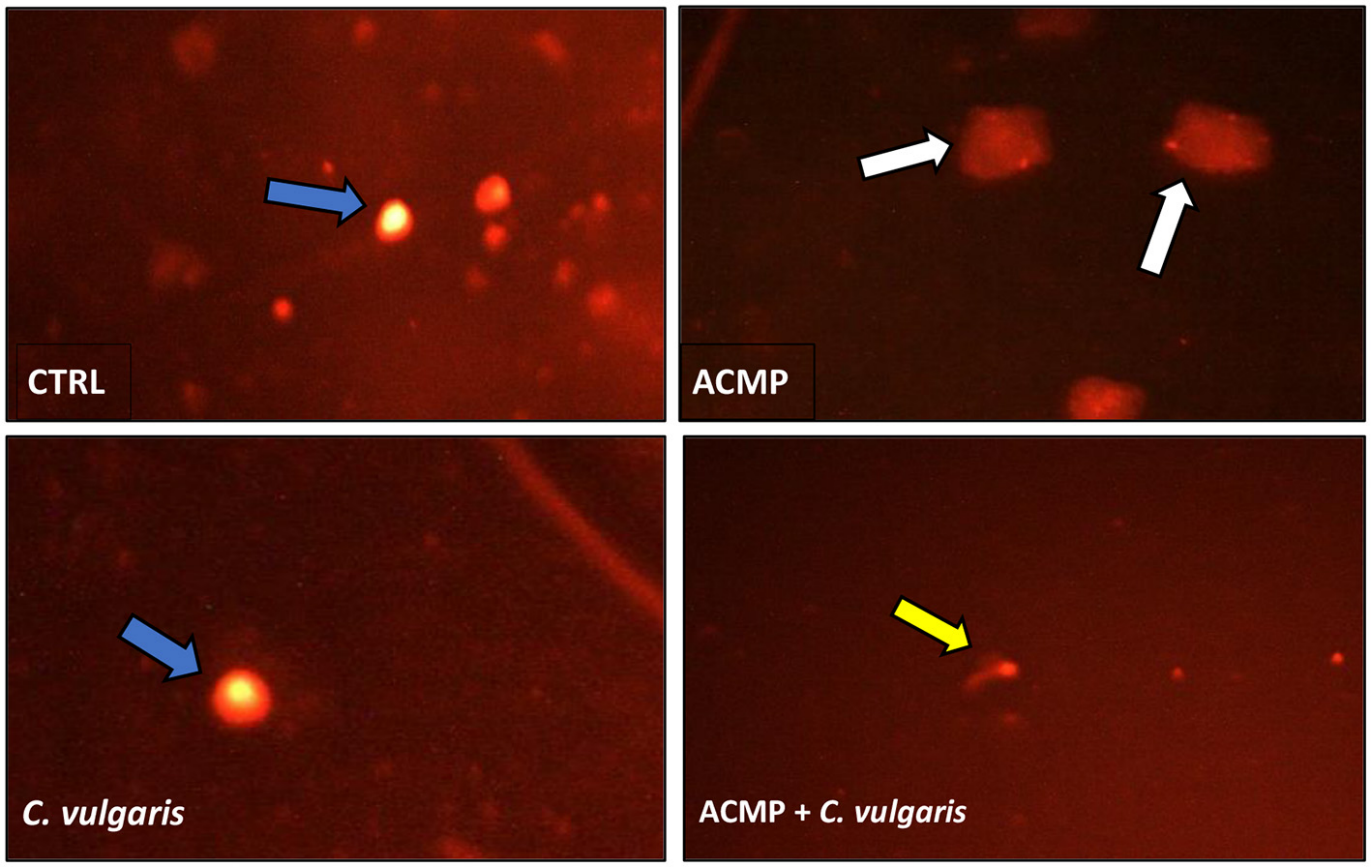
Photomicrographs represent the different degrees of DNA damage in the liver of male rats as evaluated by the comet assay after treatment by ACMP and the co-treatment of *C. vulgaris* with ACMP. Blue arrows: intact nuclei, yellow arrows: low degree of damage, white arrows: high degree of damage in different treated groups. CTRL, control; ACMP, acetamiprid; *C. vulgaris, Chlorella vulgaris*; ACMP + *C. vulgaris*, acetamiprid + *Chlorella vulgaris*. Ethidium bromide staining under ×400 of a fluorescence microscope.

Our results showed that the damage percentage, tail length (μm), DNA percentage in the tail, and tail moment in the liver of the CTRL group were 9.61 ± 0.17, 7.89 ± 0.25, 9.18 ± 0.27, and 0.77 ± 0.05, respectively. After sub-chronic exposure to ACMP, the results showed a significant (*P* < 0.05) increase in these parameters (21.34 ± 0.35, 11.92 ± 0.48, 13.78 ± 0.45, and 1.53 ± 0.07) compared to the CTRL group. However, there was a significant reduction in damage percentage, tail length (μm), DNA percentage in tail, and tail moment in ACMP + *C. vulgaris*-treated rats (12.60 ± 0.17, 8.52 ± 0.16, 10.27 ± 0.36, and 0.91 ± 0.03) compared to the ACMP group. *In C. vulgaris*-treated rats, there was a marked and significant reduction in these parameters (7.89 ± 0.17, 6.07 ± 0.15, 7.28 ± 0.16, and 0.47 ± 0.03) when compared to the CTRL group (Table 4, Figure 1).

**Table 4:** Effect of oral administration of ACMP (21.7 mg/kg BW) and/or *C. vulgaris* (150 mg/kg BW) on DNA damage in the liver of male rats (*n*/group = 8).

Experimental groups	Damage (%)	Tail length (μM)	DNA% in tail	Tail moment
CTRL	9.61 ± 0.17^c^	7.89 ± 0.25^c^	9.18 ± 0.27^c^	0.77 ± 0.05^c^
ACMP	21.34 ± 0.35^a^	11.92 ± 0.48^a^	13.78 ± 0.45^a^	1.53 ± 0.07^a^
*C. vulgaris*	7.89 ± 0.17^d^	6.07 ± 0.15^d^	7.28 ± 0.16^d^	0.47 ± 0.03^d^
ACMP + *C. vulgaris*	12.60 ± 0.17^b^	8.52 ± 0.16^b^	10.27 ± 0.36^b^	0.91 ± 0.03^b^

Within the same column, means bearing different superscripts were significant at *P* < 0.05. Results are expressed as mean ± SEM. CTRL, control; ACMP, acetamiprid; *C. vulgaris, Chlorella vulgaris*; ACMP + *C. vulgaris*, acetamiprid + *Chlorella vulgaris*.

#### 3.3.2 Testis

**Figure 2:**
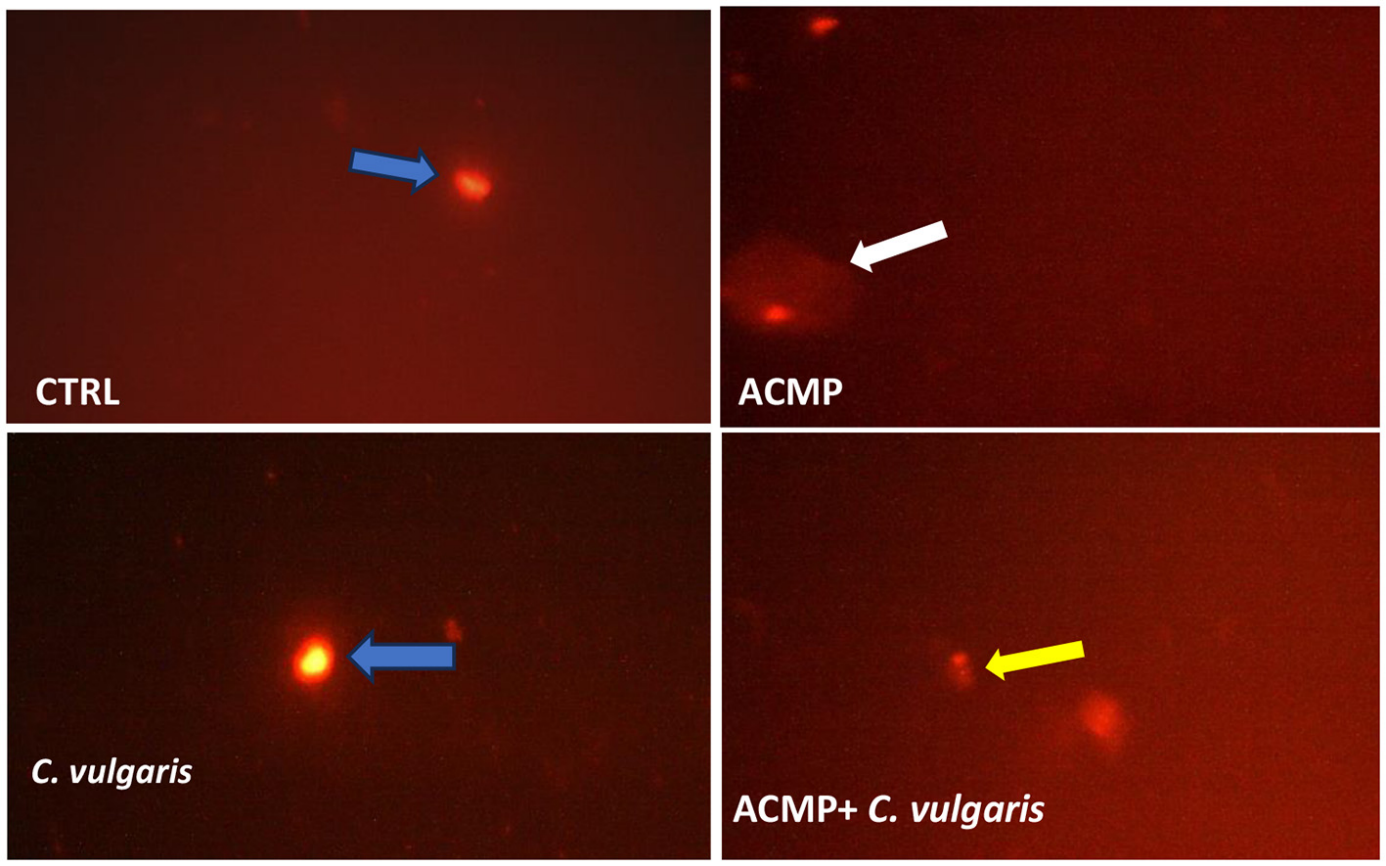
Photomicrographs representative of the different degrees of DNA damage in the testis of male rats as evaluated by the comet assay after treatment by ACMP and the co-treatment of *C. vulgaris* with ACMP. Blue arrows: intact nuclei, yellow arrows: low degree of damage, white arrows: high degree of damage in different treated groups. CTRL, control; ACMP, acetamiprid; *C. vulgaris, Chlorella vulgaris*; ACMP + *C. vulgaris*, acetamiprid + *Chlorella vulgaris*. Ethidium bromide staining under × 400 of a fluorescence microscope.

Table 5 shows the results of the comet assay in the testis. The obtained results revealed that damage percentage, tail length (μM), DNA percentage in tail, and tail moment of the CTRL group were 10.40 ± 0.23, 8.52 ± 0.35, 4.62 ± 0.33, and 0.38 ± 0.04, respectively. The ACMP group revealed a significant (*P* < 0.05) increase in damage percentage, tail length (μM), DNA percentage in tail, and tail moment (16.45 ± 0.23, 14.25 ± 0.39, 7.54 ± 0.21, and 1.44 ± 0.03, respectively) compared to the CTRL group. On the other hand, in the ACMP + *C. vulgaris* group, there was a significant (*P* < 0.05) reduction in these parameters (12.17 ± 0.19, 10.15 ± 0.17, 5.70 ± 0.21, and 0.62 ± 0.03) compared to the ACMP group. In the *C. vulgaris* group, there was a significant decrease in the same parameters (7.59 ± 0.10, 7.23 ± 0.27, 3.45 ± 0.30, and 0.22 ± 0.03) when compared to the CTRL group, as presented in Table 5 and Figure 2.

**Table 5:** Effect of oral administration of ACMP (21.7 mg/kg BW) and/or *C. Vulgaris* (150 mg/kg BW) on DNA damage in the testis of male rats (*n*/group = 8).

Experimental groups	Damage (%)	Tail length (μM)	DNA% in tail	Tail moment
CTRL	10.40 ± 0.23^c^	8.52 ± 0.35^c^	4.62 ± 0.33^c^	0.38 ± 0.04^c^
ACMP	16.45 ± 0.23^a^	14.25 ± 0.39^a^	7.54 ± 0.21^a^	1.44 ± 0.03^a^
*C. vulgaris*	7.59 ± 0.10^d^	7.23 ± 0.27^d^	3.45 ± 0.30^d^	0.22 ± 0.03^d^
ACMP + *C. vulgaris*	12.17 ± 0.19^b^	10.15 ± 0.17^b^	5.70 ± 0.21^b^	0.62 ± 0.03^b^

Within the same column, means bearing different superscripts were significant at P < 0.05. Results are presented as mean ± SEM. CTRL, control; ACMP, acetamiprid; *C. vulgaris, Chlorella vulgaris*; ACMP + *C. vulgaris*, acetamiprid + *Chlorella vulgaris*.

### 3.4 Histopathological findings

#### 3.4.1 Liver

**Figure 3:**
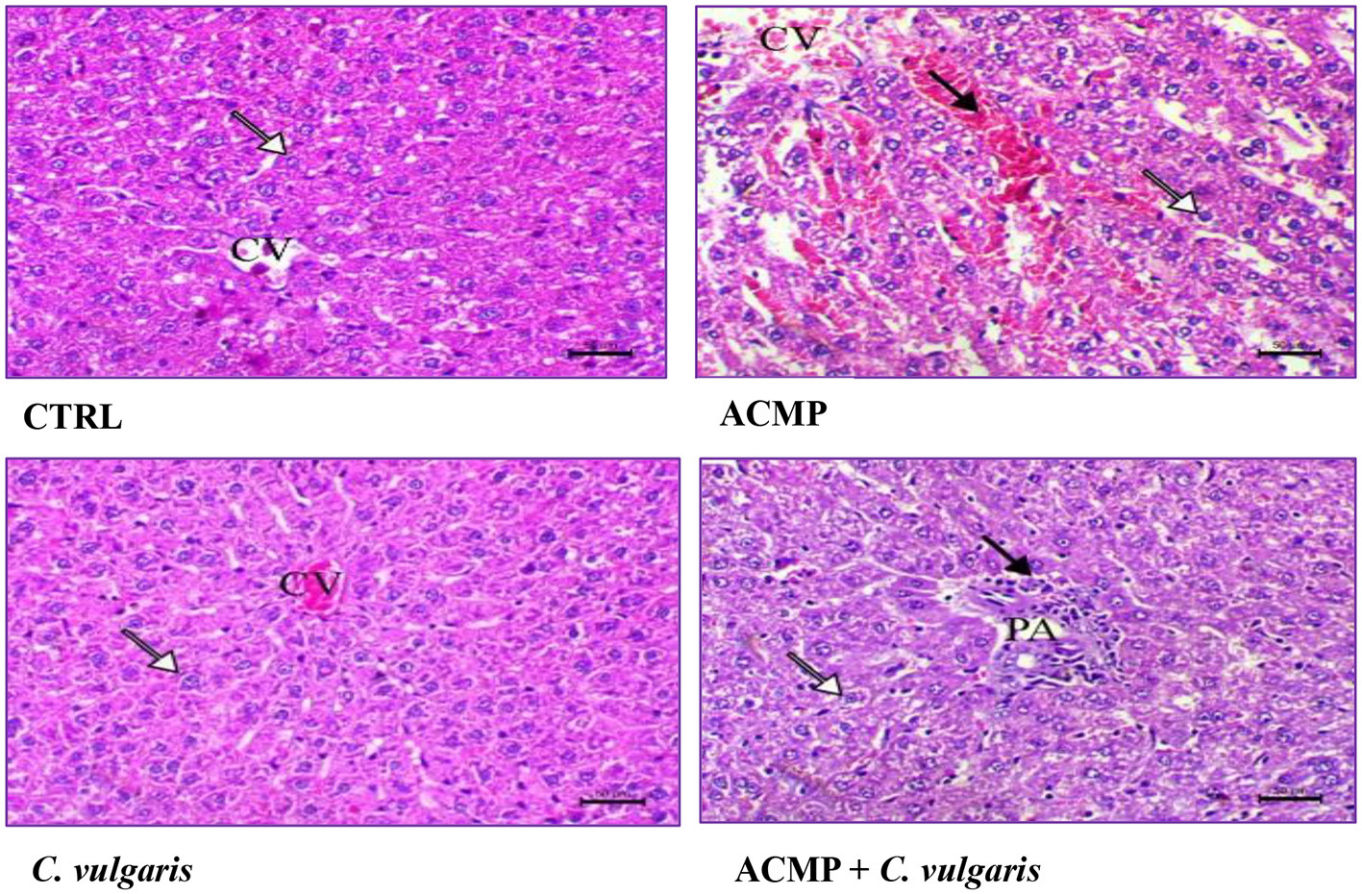
Histopathological changes in the liver of the four experimental groups. Liver of the CTRL (control) group, showing normal hepatocytes (arrow) arranged in cords around the central vein (CV). Liver of a rat intoxicated by ACMP (acetamiprid), showing marked hepatic vaculation consistent with marked hydropic changes (white arrow), and marked congestion of the central vein (CV) and hepatic sinusoids (black arrow). Liver of *C. vulgaris* (*Chlorella vulgaris*), showing normal hepatocytes (arrow) arranged around the central vein (CV). Liver of rat co-treated with ACMP + *C. vulgaris*, showing a mild degree of hepatic vacuolar changes (white arrow) and mild portal inflammation associated with mild mononuclear cells infiltration (black arrow). PA, portal area. H&E stain, × 200, bar = 50 μm.

Figure 3, CTRL revealed that the liver of the CTRL group showed that the normal hepatocytes were arranged around the central vein with normal peripheral structure between hepatic lobules, consisting of a normal portal area including the portal vein, hepatic artery, and normal bile duct. While the liver of animals intoxicated with ACMP showed severe congestion of the central vein, hepatic sinusoids, and multifocal hepatic necrosis replaced with marked mononuclear cell infiltration, including lymphocytes and macrophages (Figure 3, ACMP). Further, there was marked portal inflammation associated with disruption of hepatic parenchyma around the portal area and marked mononuclear inflammatory cell infiltration consisting of lymphocytes and macrophages, as shown in Figure 3, ACMP. Animals administered with ACMP and *C. vulgaris* showed decreased degenerative necrosis and inflammatory changes in hepatic parenchyma, and the portal area showed mild periportal inflammation with mild infiltration of mononuclear cells. Also, hepatic cells showed mild hydropic changes associated with mild cellular swelling (Figure 3, ACMP + *C. vulgaris*). The *C. vulgaris* group showed normal hepatic parenchyma, and there were no pathological alterations in this group, as shown in Figure 3, *C. vulgaris*.

#### 3.4.2 Testis

**Figure 4:**
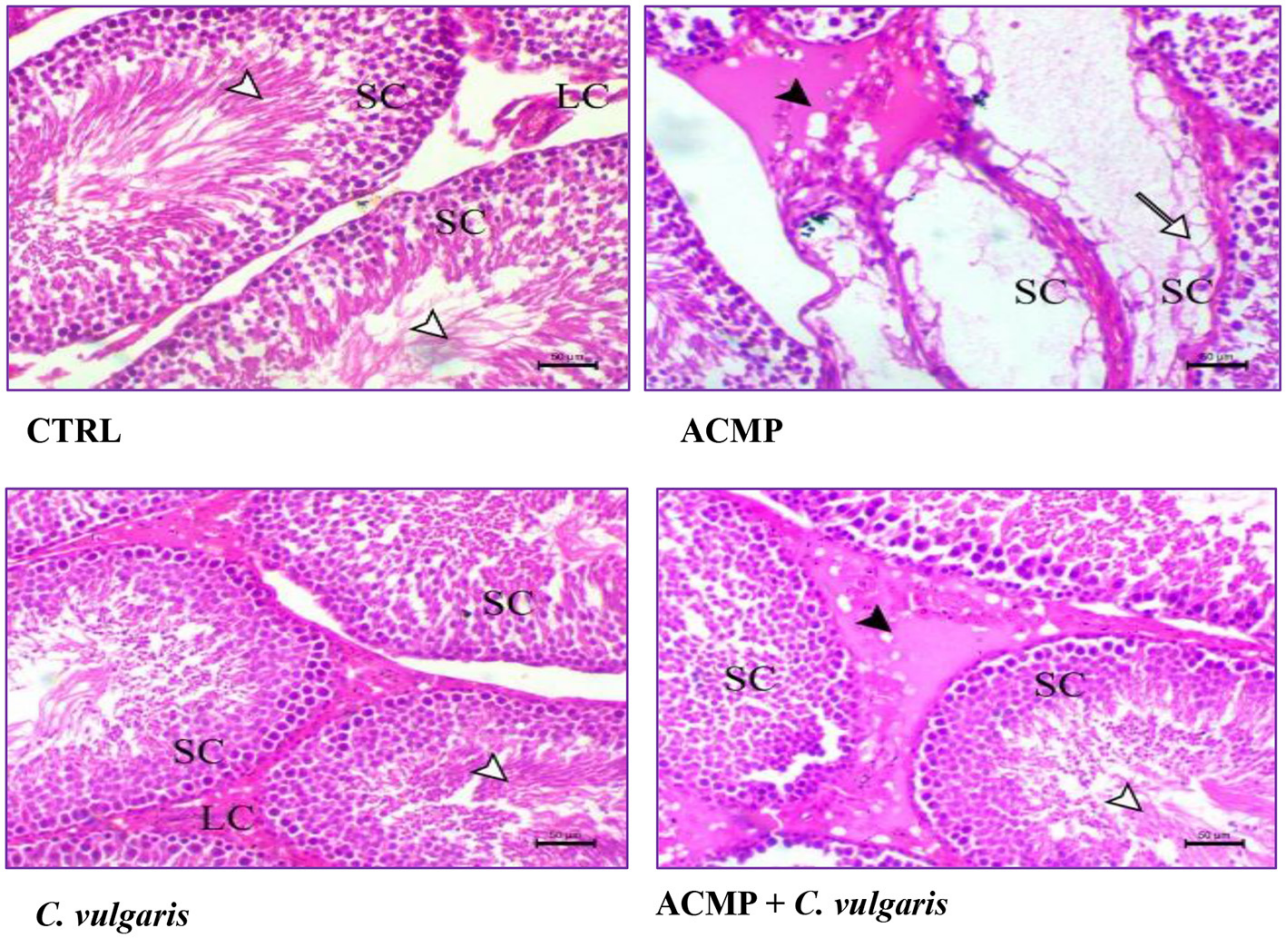
Histopathological changes in the testis of the four experimental groups. Testis of the CTRL (control) group, showing normal seminiferous tubules lined by spermatogonic cells (SC), free sperms in the lumen (white arrowhead), and normal interstitial Leydig's cells (LC). Testis of a rat intoxicated by ACMP (acetamiprid), showing a severe degree of tubular atrophy associated with complete necrosis of the spermatogonic cells (white arrow) and marked interstitial edema (black arrowhead). Testis of *C. vulgaris* (*Chlorella vulgaris*) treated group, showing normal seminiferous tubules lined by spermatogonic cells (SC), free sperms in the lumen (white arrowhead), and normal interstitial Leydig's cells (LC). Testis of rat co-treated with ACMP + *C. vulgaris*, showing normal spermatogonic cell layers (SC) with free sperms within the lumen (white arrowhead), and edema between the seminiferous tubules (black arrowhead). H&E stain, × 200, bar = 50 μm.

The testis of the CTRL group (Figure 4, CTRL) showed normal seminiferous tubules lined with normal spermatogenic cells, with the presence of free sperm cells within their lumen. The testis in the ACMP group showed testicular degeneration associated with complete necrosis of the germinal epithelium. Also, there was marked interstitial edema associated with hyperplasia of Leydig cells (Figure 4, ACMP). Co-administration of *C. vulgaris* with ACMP showed a marked decrease in the degenerative changes within the germinal epithelium, with normal spermatogenesis rather than edema present between seminiferous tubules, as shown in Figure 4, ACMP + *C. vulgaris*. Rats treated with *C. vulgaris* showed normal testicular tissue with normal germinal epithelium, as shown in Figure 4, *C. vulgaris*.

## 4 Discussion

The current study focused on the toxicity of ACMP that induced hepatic and testicular damage using a rat model, besides evaluating the extent of the protective effect of *C. vulgaris*. Therefore, in this paper, the hepatic and testicular damage induced by ACMP was evaluated by examination of changes in sperm characteristics, besides measurement of oxidant and antioxidant parameters, DNA damage, and histopathological changes in hepatic and testicular tissues.

Sperm motility, viability, plasma membrane integrity, and sperm cell count are all critical parameters that influence fertility (55). Our results showed a significant decline in sperm characteristics, including motility, plasma membrane integrity, viability, and SCC of the ACMP group in comparison to the CTRL group. This was in concordance with the findings of Mosbah et al. (56) and Augustave et al. (57) in rats and guinea pigs, respectively, which treated with ACMP. This decrease in sperm quality could be a result of the plasma membrane disintegration, motility loss, and sperm cell malfunction caused by ACMP-induced overproduction of ROS. Indeed, sperm cells are sensitive to ROS because their plasma membrane includes large quantities of PUFAs; consequently, excessive ROS generation in semen by leukocytes may be the cause of low sperm quality (58, 59).

However, there was a significant amelioration in sperm characteristics, including motility, plasma membrane integrity, viability, and SCC in *C. vulgaris* co-administered with ACMP-treated rats when compared to the ACMP group. This improvement may be attributed to the antioxidant properties of *C. vulgaris*, which directly enhance spermatogenesis and sperm quality (60). The improvement in the sperm count owing to *C. vulgaris* was attributed to the enhanced testicular development or accelerated spermatogenesis (61), and the improvement of the sperm motility was attributed to the modification of the spermatozoa or the seminal plasma content of PUFAs (62), which have been shown to support the flagellar activity and bending movement (62). In this aspect, *C. vulgaris* may be preferable to oils because it includes both PUFAs and antioxidants (34). These findings were in line with Murphy et al. (62), who mentioned that the SCC and motility were shown to be improved by a supplement containing a source of PUFAs, such as a commercial algal product. Moreover, these findings were consistent with those of earlier research by Osama et al. (60), who studied the protective effect of *C. vulgaris* (50 mg/kg BW) on deltamethrin-induced toxicity in male rats. They found that there was an increase in sperm count, motility, and viability in the co-treatment group when compared to the deltamethrin group.

Oxidative stress is considered to be a main component of the mechanism of ACMP toxicity; hence, assessing oxidative stress biomarkers is critical. In this regard, we studied the effect of ACMP on MDA, as an LPO marker, and on antioxidant enzymes, including CAT, SOD, and GSH. Our data displays a statistically significant increase in the level of MDA in the liver and testis of rats treated with ACMP when compared to the CTRL group. MDA, as an indication of LPO, is elevated during oxidative stress, indicating the degree of LPO, and decreases when the antioxidant defense system is activated (63). ACMP-produced free radicals bind covalently to protein and DNA, resulting in oxidative damage such as LPO, protein degradation, and DNA damage (64). Oxidative stress and overproduction of ROS by ACMP may be associated with the increase of MDA concentration in the liver and testis, which was consistent with previous publications (14, 19, 41).

On the contrary, our findings showed a significant decrease in CAT, SOD, and GSH activities in hepatic and testicular tissues of the ACMP group in comparison to the CTRL group. These results indicate the presence of oxidative stress in these tissues. Thus, it corroborates the fact that ACMP-induced reproductive and hepatic toxicity was mediated through an oxidative stress mechanism (19, 65). These results are in accordance with previous studies that demonstrated oxidative stress in hepatic tissue following exposure to 21.7 mg/kg BW (41) ACMP or 20 mg/kg BW (66) ACMP due to ROS overproduction and impairment in the antioxidant defense system.

While co-administration of *C. vulgaris* with ACMP showed a significant decrease in MDA and a significant increase in CAT, SOD, and GSH levels when compared to the ACMP group. It is well known that *C. vulgaris* is a natural source of antioxidants, including flavonoids, carotenoids, vitamins, and minerals. It can act as a free radical scavenger, reducing oxidative damage in cells (67). This result supports the finding of Ranjbar et al. (68), who found that *C. vulgaris* co-treated with carbon tetrachloride decreased MDA and increased SOD and GSH in rats compared to the carbon tetrachloride group only. Similarly, *C. vulgaris* supplementation significantly elevated the antioxidant status of the body and the milk in goats (69).

Most pesticides are assessed for mutagenicity, which includes DNA damage. Oxidative stress can cause DNA damage (70). Insecticides have been linked to several forms of DNA damage, such as base-free sites, base modifications, deletions, frame shifts, and chromosomal rearrangements, due to the insecticides' artificial production of ROS (71). Our study found a significant increase in DNA damage in the ACMP group compared to the CTRL group, which was measured by comet parameters such as damage percentage, tail length, DNA percentage in tail, and tail moment in hepatic and testicular tissues. This could be attributed to oxidative stress and decreased antioxidant levels in ACMP-treated rats.

Çavaş et al. (72) found that ACMP exposure resulted in micronucleus production and DNA damage in Caco-2 cells. Kammoun et al. (73) reported extensive DNA damage in the testicular tissue of thiacloprid-treated rats. DNA fragmentation was also seen in rat testicular tissue following imidacloprid exposure in immature and mature rats (74). In the same context, previous studies demonstrated that oxidative stress is the mechanism by which imidacloprid induces reproductive damage by reporting elevated MDA, LPO marker, and declined CAT, SOD, GSH, and GPx levels in male rats (2, 75, 76). This means that ACMP had a genotoxic effect that damaged DNA, which agreed with previous studies due to all the previous compounds belonging to the same group, neonicotinoid insecticides. However, co-administering *C. vulgaris* with ACMP showed a significant decrease in DNA damage when compared to ACMP-treated rats. This may be due to the antioxidant property of *C. vulgaris*. This result agrees with Ranjbar et al. (68), who recorded that *C. vulgaris* decreased DNA fragmentation induced by carbon tetrachloride in the rat sperm cells.

Upon examining hepatic and testicular histopathology, the present study found that ACMP induced several alterations in hepatic and testicular tissues. In the liver, there is severe congestion of the central vein, hepatic sinusoids, and multifocal hepatic necrosis replaced with marked mononuclear cell infiltration, including lymphocytes and macrophages, which agrees with previous studies (41, 77). In the testis, testicular degeneration was associated with complete necrosis of the germinal epithelium. There was marked interstitial edema associated with hyperplasia of Leydig cells. Rats co-treated with *C. vulgaris*, though, displayed normal histopathological findings in both the liver and testis, almost similar to the CTRL rats.

Although we found greater antioxidant activities (CAT, SOD, and GSH) and lower oxidant activity (MDA) in hepatic and testicular tissues collected from the *C. vulgaris* group. The lack of the biological mechanism of *C. vulgaris* as a potent antioxidant is considered the main limitation of our trial. *C. vulgaris* is well-known for being rich in important nutrients such as proteins, vitamins, minerals, and bioactive components, including carotenoids (lutein and β-carotene), chlorophyll, polyunsaturated fatty acids (PUFAs), polyphenols, and polysaccharides, all of which contribute to its several medicinal benefits (78). These chemicals have been shown to have antioxidant, anti-inflammatory, immunomodulatory, and detoxifying effects (79).

Antioxidants work in two ways: non-enzyme-promoted and enzyme-promoted antioxidants. Non-enzyme-promoted antioxidants work directly by neutralizing free radicals, but enzyme-promoted antioxidants strengthen the body's natural antioxidant defenses by increasing the activity of critical enzymes responsible for ROS breakdown. Non-enzyme-promoted antioxidants such as Chlorophyll, an essential component of *C. vulgaris*, have been demonstrated to scavenge free radicals. Enzyme-promoted antioxidants strengthen the body's natural antioxidant defenses by increasing the activity of critical enzymes responsible for ROS breakdown, such as CAT and SOD. These enzymes play an important role in ROS neutralization by transforming superoxide radicals into harmless compounds. The anti-inflammatory effects of *C. vulgaris* are strong due to its bioactive constituents, including carotenoids and polysaccharides. These chemicals reduce the synthesis of pro-inflammatory cytokines, including tumor necrosis factor-α (TNF-α) and interleukin-6 (IL-6), and decrease the activity of inflammatory enzymes like Cyclooxygenase-2 (COX-2) (80). Clarification of the mechanistic roles of these chemicals in the antioxidant effect of *C. vulgaris* requires further investigation in our future studies.

## 5 Conclusions

In conclusion, our study showed the protective effect *of C. vulgaris* against ACMP-induced hepatic and testicular toxicity in rats, which is mediated through attenuation of ACMP-induced oxidative stress and improved sperm characteristics, hepatic, and testicular oxidant/antioxidant status. Therefore, *C. vulgaris* may be recommended as a food additive to improve hepatic and testicular function under normal and oxidative stress conditions. Re-evaluation of this effect in an economically important animal species is highly recommended and suspected to be of valuable practical application.

## Data Availability

The original contributions presented in the study are included in the article/supplementary material, further inquiries can be directed to the corresponding author/s.
